# Editorial: How can corneal biomechanics help with clinical applications?

**DOI:** 10.3389/fbioe.2023.1184840

**Published:** 2023-04-12

**Authors:** FangJun Bao, WeiYi Chen, Yan Wang, Ahmed Elsheikh

**Affiliations:** ^1^ Eye Hospital, Wenzhou Medical University, Wenzhou, China; ^2^ College of Biomedical Engineering, Taiyuan University of Technology, Taiyuan, China; ^3^ Tianjin Eye Hospital, Tianjin, China; ^4^ School of Engineering, University of Liverpool, Liverpool, United Kingdom

**Keywords:** cornea, biomechanics, *ex-vivo* measurement, *in-vivo* measurement, clinical application

For those of us on the corneal biomechanics bandwagon, the 25-year journey to date has been challenging and yet extremely exciting. Although interest in the field goes back to the 1970s and before, serious interest from renowned researchers like Woo, Wollensak, Nash and Bryant started to make significant contributions in the 1990s. At that time, most, if not all, efforts were focused on the *ex-vivo* testing of corneal tissue in an attempt to understand and quantify the material’s hyperelasticity, viscoelasticity and hysteresis, and the behaviour changes associated with ageing and diseases such as diabetes and keratoconus. Most of that work used uniaxial extensometry testing, with narrow strips separated from corneal tissue (and surrounding limbus and anterior sclera) and subjected to tension forces. The tests suffered from fundamental drawbacks arising from the specimen’s non-uniform thickness, straightening an originally curved tissue and the severing of collagen fibrils along specimen edges. Furthermore, the loading applied (axial tension) was not compatible with the usual loading of the cornea under intraocular pressure.

Efforts to improve the testing protocol led to cornea inflation testing, in which the cornea remained intact and loaded with a posterior pressure simulating closely the effects of intraocular pressure. Still, the test method relied on non-physiologic boundary conditions along the specimen edge, which did not represent the actual stiffness of the limbus. In addition, approximations were also introduced in the analysis of the pressure-deformation results to produce estimates of the stress-strain behaviour; this analysis relied first on a form of shell theory that involved necessary approximations of the cornea as a spherical segment with rotational symmetry and uniform thickness. These features inevitably affected the reliability of the resulting stress-strain behaviour and underscored the need for improvements in the test method.

Improvements came first in the form of the finite element-based inverse analysis of the cornea inflation test results–removing the need for shell theory. A more substantial improvement followed with whole globe inflation testing, where complete intact eye globes were tested under variable intraocular pressure while suspending the eye and monitoring its deformation response to pressure changes. This technique presented the ultimate solution as the tissue remained intact, loaded with physiologic intraocular pressure, and no approximations were made in the analysis method; however, the drawback was in the complexity of the testing protocol and the associated high chance of analysis mistakes. The protocol was also very time consuming and required the development of highly complex testing apparatus, access to which was limited to a small number of research groups around the world. Despite this, the resulting information on corneal biomechanical behaviour was invaluable in quantifying trends related to stiffness changes with age, disease and rate of loading.

The results also showed that corneas with the same conditions (age, race, medical history) still experienced different behaviours. This outcome meant that, while *ex-vivo* testing helped identify corneal behaviour trends, these trends could not simply be applied to individual corneas. Another Research Topic arose with the realisation–or expectation–that *ex-vivo* tissue may have undergone deterioration post-mortem, and therefore its experimental behaviour cannot be assumed to apply directly to *in vivo* eyes. For these reasons, it became evident that *in vivo* testing and measurement of tissue biomechanics was a necessary next step. The first breakthrough here was in 2005 with the Ocular Response Analyzer (ORA, Reichert, USA), a non-contact air-puff-based instrument that provided two measures of corneal biomechanics; these are Corneal Resistance Factor (CRF), which relates to corneal geometric stiffness–in turn, dominated by changes in corneal thickness–and Corneal Hysteresis (CH), which relates to corneal viscoelasticity. This instrument had a significant impact on the field by showing promise in the *in vivo* measurement of corneal biomechanics, even though the CRF and CH could not be linked directly to standard measures of stiffness such as the tangent modulus.

A subsequent development took place a few years later, in 2012, with the Corvis ST (Oculus, Germany). This device used similar non-contact, air puff technology and used its much-enhanced monitoring technique to derive several dynamic corneal response (DCR) parameters. Some of the DCRs, most notably the Stiffness Parameter (SP), the Integrated Inverse Radius (IIR), and the Deflection Amplitude (DA) could be related statistically to corneal biomechanical stiffness, even though (like the CRF and CH) no direct links between them and the standard stiffness measures could be established. A further development then took place in 2018 with the Stress Strain Index (SSI), which promised to estimate the stress-strain behaviour of the tissue and, hence, its tangent modulus at any stress or strain. This development was taken further 2 years later when it became possible to turn the SSI from a single value to a map covering the corneal surface. This step was made possible by relying on the evident link between corneal stiffness and the density and orientation of the tissue’s collagen fibrils, as well as our current knowledge of the tissue’s microstructure maps of both healthy and diseased corneas.

Other *in vivo* behaviour testing methods are under development and expected to become commercially available within a few years. Most notably, Brillouin Microscopy and elastography are among the most detailed and most advanced techniques with strong scientific evidence of their ability to map corneal stiffness in 3D.

As we now move into the second quarter of the 21st century, the wealth of knowledge on ‘trends’ of corneal behaviour identified in *ex-vivo* tests, and ‘quantification and mapping’ of the behaviour of individual corneas measured *in vivo*, is allowing us to move into treatment optimisation for the first time. This area is the ultimate goal of all our previous efforts as a research community over the last 25 years. And it is exciting because now the target is to benefit patients, rather than to develop methods or understand behaviour. Several applications in which clinicians have had to rely on experience can now be optimised on a scientific basis and with reference to accurate measurements of corneal biomechanics *in vivo*. These applications include the optimisation of the crosslinking treatment used in patients with keratoconus, customisation of the intracorneal ring segment surgeries used to restore the healthy geometry in irregular corneas, and the incisions used in cataract surgeries which may have an effect on corneal astigmatism.

These applications and others are expected to provide bright examples of how corneal biomechanics can really benefit clinical practice over the next few years. So, watch the space!

